# Supplementary material for: Empirical methods for controlling false positives and estimating confidence in ChIP-Seq peaks
Source: BMC Bioinformatics. 2008 Dec 5;9:523. doi: 10.1186/1471-2105-9-523 (PMC2628906; doi:10.1186/1471-2105-9-523)
Supplement: Additional File 1 — A variety of html documents from the USeq web site detailing the available applications, their best usage, output file type descriptions, command line menus, etc. [file 1471-2105-9-523-S1.zip › USeqUserGuides/JavaDoc.zip!/JavaDoc/util/bio/digest/class-use/Enzyme.html]

Uses of Class util.bio.digest.Enzyme


|  |  |  |
| --- | --- | --- |
| **Overview** \| **Package** \| **Class** \| **Use** \| **Tree** \| **Deprecated** \| **Index** \| **Help** | |  |
| PREV   NEXT | **FRAMES**    **NO FRAMES**     **All Classes** |  |


---


## **Uses of Class util.bio.digest.Enzyme**

| Packages that use Enzyme | |
| --- | --- |
| **bioroot.oligo** |  |
| **bioroot.plasmid** |  |
| **util.bio.digest** | Restriction enzyme digestion of DNA sequences. |

Uses of Enzyme in bioroot.oligo

| Fields in bioroot.oligo declared as Enzyme | |
| --- | --- |
| `static Enzyme[]` | `OligoBase.ENZYMES` |

| Methods in bioroot.oligo with parameters of type Enzyme | |
| --- | --- |
| `java.lang.String` | `OligoBean.getRestrictionCutSites(Enzyme[] enzymes)` |

Uses of Enzyme in bioroot.plasmid

| Methods in bioroot.plasmid with parameters of type Enzyme | |
| --- | --- |
| `java.lang.String` | `PlasmidBean.getRestrictionCutSites(Enzyme[] enzymes)` |

Uses of Enzyme in util.bio.digest

| Methods in util.bio.digest that return Enzyme | |
| --- | --- |
| `Enzyme[]` | `NEBParser.getEnzymes()` |
| `Enzyme[]` | `NEBParser.makeEnzymeArray(java.lang.String gtype2c, int minLengthRecogSeq)` |
| `static Enzyme[]` | `REDigest.restrictionMap(java.lang.String DNA, Enzyme[] e)`             Runs thru an enzyme array attempting to cut a particular sequence |

| Methods in util.bio.digest with parameters of type Enzyme | |
| --- | --- |
| `static java.lang.String` | `REDigest.getRestrictionMapString(java.lang.String seq, Enzyme[] enzymes)` |
| `static Enzyme[]` | `REDigest.restrictionMap(java.lang.String DNA, Enzyme[] e)`             Runs thru an enzyme array attempting to cut a particular sequence |
| `static java.util.ArrayList` | `REDigest.restrictionMapHTML(java.lang.String DNA, Enzyme[] e)`             Returns an arraylist of enzymes found to cut the sequence |
| `static java.lang.String` | `REDigest.restrictionMapList(java.lang.String DNA, Enzyme[] e)`             Restriction map a sequence. |

---


|  |  |  |
| --- | --- | --- |
| **Overview** \| **Package** \| **Class** \| **Use** \| **Tree** \| **Deprecated** \| **Index** \| **Help** | |  |
| PREV   NEXT | **FRAMES**    **NO FRAMES**     **All Classes** |  |


---
